# Patient Characteristics and Clinical Course of COVID-19 Patients Treated at a German Tertiary Center during the First and Second Waves in the Year 2020

**DOI:** 10.3390/jcm10112274

**Published:** 2021-05-24

**Authors:** Thomas Theo Brehm, Andreas Heyer, Kevin Roedl, Dominik Jarczak, Axel Nierhaus, Michael F Nentwich, Marc van der Meirschen, Alexander Schultze, Martin Christner, Walter Fiedler, Nicolaus Kröger, Tobias B Huber, Hans Klose, Martina Sterneck, Sabine Jordan, Benno Kreuels, Stefan Schmiedel, Marylyn M Addo, Samuel Huber, Ansgar W Lohse, Stefan Kluge, Julian Schulze zur Wiesch

**Affiliations:** 1 I. Department of Internal Medicine, University Medical Center Hamburg-Eppendorf, Martinistraße 52, 20246 Hamburg, Germany; andreas.heyer@stud.uke.uni-hamburg.de (A.H.); mvdmeirschen@gmail.com (M.v.d.M.); sterneck@uke.de (M.S.); s.jordan@uke.de (S.J.); b.kreuels@uke.de (B.K.); s.schmiedel@uke.de (S.S.); m.addo@uke.de (M.M.A.); s.huber@uke.de (S.H.); a.lohse@uke.de (A.W.L.); j.schulze-zur-wiesch@uke.de (J.S.z.W.); 2 German Center for Infection Research (DZIF), Partner Site Hamburg-Lübeck-Borstel-Riems, Germany; 3 Department of Intensive Care Medicine, University Medical Center Hamburg-Eppendorf, Martinistraße 52, 20246 Hamburg, Germany; k.roedl@uke.de (K.R.); d.jarczak@uke.de (D.J.); nierhaus@uke.de (A.N.); m.nentwich@uke.de (M.F.N.); s.kluge@uke.de (S.K.); 4 Department of Emergency Medicine, University Medical Center Hamburg-Eppendorf, Martinistraße 52, 20246 Hamburg, Germany; a.schultze@uke.de; 5 Institute of Medical Microbiology, Virology and Hygiene, University Medical Center Hamburg-Eppendorf, Martinistraße 52, 20246 Hamburg, Germany; m.christner@uke.de; 6 Department of Oncology, Hematology and Bone Marrow Transplantation with Section Pneumology, University Medical Center Hamburg-Eppendorf, Martinistraße 52, 20246 Hamburg, Germany; fiedler@uke.de (W.F.); klose@uke.de (H.K.); 7 Department of Stem Cell Transplantation, University Medical Center Hamburg-Eppendorf, 20246 Hamburg, Germany; n.kroeger@uke.de; 8 III. Department of Medicine, University Medical Center Hamburg-Eppendorf, 20246 Hamburg, Germany; t.huber@uke.de

**Keywords:** SARS-CoV-2, COVID-19, patients, hospital, mortality, first wave, second wave, treatment

## Abstract

In this study, we directly compared coronavirus disease 2019 (COVID-19) patients hospitalized during the first (27 February–28 July 2020) and second (29 July–31 December 2020) wave of the pandemic at a large tertiary center in northern Germany. Patients who presented during the first (*n* = 174) and second (*n* = 331) wave did not differ in age (median [IQR], 59 years [46, 71] vs. 58 years [42, 73]; *p* = 0.82) or age-adjusted Charlson Comorbidity Index (median [IQR], 2 [1, 4] vs. 2 [0, 4]; *p* = 0.50). During the second wave, a higher proportion of patients were treated as outpatients (11% [*n* = 20] vs. 20% [*n* = 67]), fewer patients were admitted to the intensive care unit (43% [*n* = 75] vs. 29% [*n* = 96]), and duration of hospitalization was significantly shorter (median days [IQR], 14 [8, 34] vs. 11 [5, 19]; *p* < 0.001). However, in-hospital mortality was high throughout the pandemic and did not differ between the two periods (16% [*n* = 27] vs. 16% [*n* = 54]; *p* = 0.89). While novel treatment strategies and increased knowledge about the clinical management of COVID-19 may have resulted in a less severe disease course in some patients, in-hospital mortality remained unaltered at a high level. These findings highlight the unabated need for efforts to hamper severe acute respiratory syndrome coronavirus type 2 (SARS-CoV-2) transmission, to increase vaccination coverage, and to develop novel treatment strategies to prevent mortality and decrease morbidity.

## 1. Introduction

After the coronavirus disease 2019 (COVID-19) emerged in Wuhan, China in December 2019, it rapidly spread worldwide and was declared a pandemic by the WHO on 11 March 2020 [1]. In Germany, the first case was confirmed at the end of January [2] and the first case in the state of Hamburg occurred at the end of February 2020 [3]. During the first wave in Germany, COVID-19 case numbers peaked in March and April 2020, when more than 4500 infections per day were reported [4]. This was followed by a progressive decrease with only few infections during the summer months and a renewed increase in October and almost 30,000 daily SARS-CoV-2 infections at the end of December 2020 [5]. This second wave was even usurped by a third wave in early 2021 [6]. Changes in transmission patterns, different public health interventions [7], and implementation of novel treatment strategies [8] may have all affected demographic characteristics as well as the disease outcome of patients hospitalized with COVID-19 during the evolving pandemic. Real-world data comparing patients hospitalized in the same clinical setting during the different stages of the pandemic are scarce. We aimed to systematically compare demographic data, comorbidities, medication, disease severity, and mortality of COVID-19 patients treated at the University Medical Center Hamburg-Eppendorf during the first and second waves of the pandemic.

## 2. Materials and Methods

### 2.1. Study Design

Previously, we presented the results of a direct comparison between a cohort of patients with seasonal influenza and the first 166 patients hospitalized with COVID-19 at our tertiary care center [9]. In this present retrospective observational study, we included all patients who presented at the University Medical Center Hamburg-Eppendorf with SARS-CoV-2 infections confirmed by reverse transcription-polymerase chain reaction (RT-PCR) in the year 2020. The beginning of the second wave was defined as the first day on which the 7-day incidence again exceeded 5 per 100,000 inhabitants in the German city-state of Hamburg [10]. Hence, patients diagnosed between 27 February and 28 July 2020, were considered to belong to the first wave of the pandemic, and patients diagnosed between 29 July and 31 December 2020 to the second wave of the pandemic. The study was reviewed and approved by the Ethics Committee of the Medical Council of Hamburg (WF-052/20). Patients younger than 18 years and hospital employees diagnosed with SARS-CoV-2 infections by our institution’s screening efforts but who did not require hospitalization were not included in the study. We further excluded patients who had already recovered from COVID-19 and presented to the hospital with conditions related or unrelated to the previous infection.

### 2.2. RT-PCR

RT-PCR for confirmation of SARS-CoV-2 infection was performed using swab samples from the upper respiratory tract or specimens from the lower respiratory tract using either a laboratory-developed test for the NeuMoDx 96 system (NeuMoDx inc., Ann Arbor, USA; distributed by QIAGEN) [11], a Cobas6800 IVD (Roche Diagnostics, Basel Switzerland), a GeneXpert Xpress System (Cepheid, Sunnyvale, CA, USA), or a Cobas6800-based UCT (Ann Arbor, MI, USA; distributed by QIAGEN) [12]. A total of 18 patients had only tested positive for SARS-CoV-2 before hospital admission but did not test positive at our institution.

### 2.3. Clinical Data

Demographic information, clinical characteristics, and disease outcomes of COVID-19 patients were extracted from patient files and stored in Microsoft Excel, version 16 for macOS (Microsoft, Seattle, WA, USA). The age-adjusted Charlson Comorbidity Index (ACCI) was calculated for each patient to assess the overall comorbidity status [13,14]. Both the median age and the number of patients by different age groups were compared between the cohorts. The final analysis of hospital course and in-hospital mortality was performed on 31 January 2020. At this time, all patients diagnosed with COVID-19 until 31 December 2020 had been discharged from the hospital, so information on disease outcome and duration of hospitalization was available for the entire cohort. Disease severity was categorized into mild, moderate, severe, and critical cases based on the criteria proposed by the WHO [15]. No systematic outpatient follow-up examinations were performed, and only in-hospital morbidity and mortality were assessed. Individuals treated as outpatients at our department of emergency medicine and deceased patients were not included in the analysis of the duration of hospitalization. For patients who contracted nosocomial SARS-CoV-2 infections, the duration of hospitalization was calculated from the day of infection.

### 2.4. Statistical Analyses

Continuous variables are expressed as the median and interquartile range (IQR) and were compared with the Mann–Whitney U test. Categorical variables are expressed as number (%) and were compared by Fisher′s exact test. A multivariate logistic regression model was used to determine whether presentation during the first versus second wave was an independent predictor for in-hospital mortality. An *a priori* decision was made to include the variables age ≥ 60 years, male sex, and ACCI in the model since these variables have been previously shown to be important risk factors for mortality in COVID-19 patients [16]. A log-rank test was used to compare the proportion of patients discharged alive during the first 120 days of hospitalization. *p* values less than 0.05 were considered statistically significant. Statistical analysis was performed using SPSS, version 26 (IBM Corp., Armonk, NY, USA). Figures were designed using GraphPad Prism, version 9 for macOS (GraphPad Software, La Jolla, CA, USA).

## 3. Results

### 3.1. Number of Patients and Seasonal Distribution

**Figure 1:**
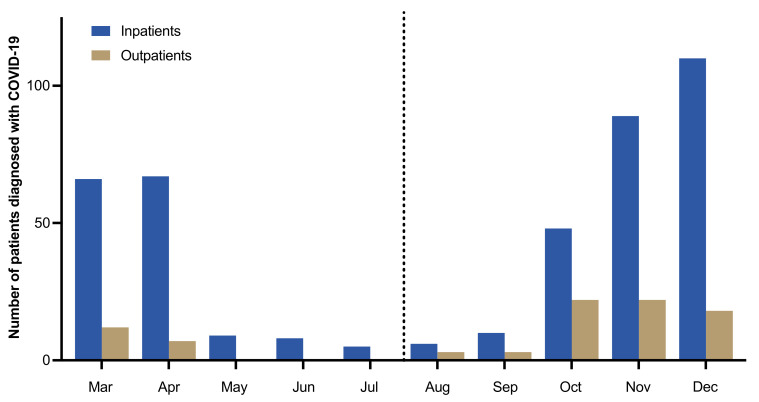
The numbers of COVID-19 patients newly admitted to our hospital or seen as outpatients in our emergency department are listed for each month. The dotted line represents the border between the first and the second wave (29 July 2020).

The University Medical Center Hamburg Eppendorf serves a catchment area of more than 4 million inhabitants of northern Germany and has more than 1400 regular care and more than 130 intensive care beds. In the year 2020, a total of 505 patients with RT-PCR confirmed COVID-19 presented to our center or were transferred from other hospitals. Of those patients, 174 presented during the first wave between 27 February and 28 July 2020, and 331 presented during the second wave between 29 July and 31 December 2020. The seasonal distribution of outpatients seen at our department of emergency medicine and patients admitted to our hospital is shown in Figure 1. The first wave peaked in April with a total of 67 hospital admissions. This was followed by a steady decrease in patient numbers and only few COVID-19 patients presented at our hospital between May and September. Case numbers again steadily increased in October and again peaked in December, with a total of 110 hospital admissions and 18 patients seen as outpatients.

### 3.2. Demographic Information and Comorbidities

**Figure 2:**
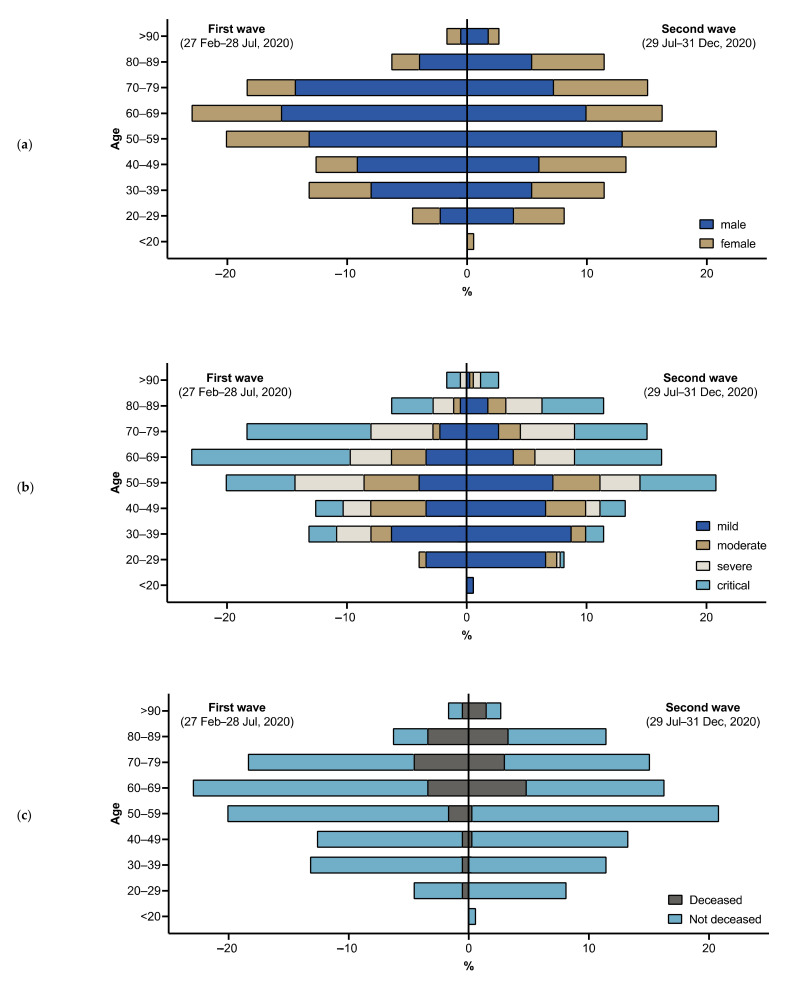
(**a**) Sex distribution, (**b**) disease severity, and (**c**) and in-hospital mortality of COVID-19 patients at our center during the first wave (left) and second wave (right) of the pandemic stratified by age group.

The proportion of male patients was significantly lower during the second wave compared to the first wave (67% [*n* = 117] vs. 53% [*n* = 175]; *p* = 0.002) (Table 1 and Figure 2a). The median age among all COVID-19 patients (median age [IQR], 59 years [46, 71] vs. 58 years [42, 73]; *p* = 0.82) as well as among those admitted to the ICU (median age [IQR], 64 years [55, 74] vs. 64 years [55, 75]; *p* = 0.99) did not differ between the two periods. However, more COVID-19 patients older than 80 years presented at our hospital during the second wave (8% [*n* = 14] vs. 14% [*n* = 47]; *p* = 0.04). The median ACCI (median [IAR], 2 [1, 4] vs. 2 [0, 4]; *p* = 0.50) did not significantly differ between the first and the second wave. Moreover, the prevalence of different comorbidities was similar among patients presenting during either of the two phases of the pandemic (Supplementary Materials Figure S1). The number of immunosuppressed individuals did not differ between the two periods (24% [*n* = 41] vs. 20% [*n* = 67]; *p* = 0.42). More detailed information on the immunosuppressed patients is shown in Supplementary Materials Table S1.

### 3.3. Course of Disease

**Figure 3:**
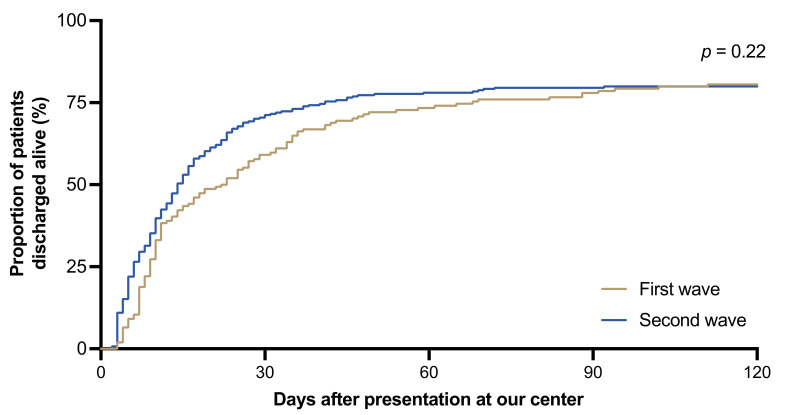
The proportion of patients discharged alive during the first and second waves compared by the log-rank test.

The number of patients presenting with a mild disease course was higher (24% [*n* = 41] vs. 39% [*n* = 128]), and the number of patients with severe (22% [*n* = 38] vs. 16% [*n* = 54]) or critical disease (39% [*n* = 68] vs. 30% [*n* = 100]) was lower during the second wave compared to the first wave of the pandemic (Table 2 and Figure 2b). During the second wave, more patients were treated as outpatients at our emergency department and not admitted to the hospital (11% [*n* = 20] vs. 20% [*n* = 67]) and fewer patients had to be admitted to the intensive care unit (43% [*n* = 75] vs. 29% [*n* = 96]). In addition, fewer patients required mechanical ventilation (32% [*n* = 56] vs. 20% [*n* = 67]; *p* = 0.004), vasopressor treatment (34% [*n* = 60] vs. 23% [*n* = 76]; *p* = 0.006), or renal replacement therapy (21% [*n* = 47] vs. 14% [*n* = 47]; *p* = 0.045). The rate of nosocomial infections significantly decreased throughout the observation period (14% [*n* = 24] vs. 1% [*n* = 4]; *p* < 0.001). The duration of hospitalization among patients admitted to our hospital was shorter during the second compared to the first wave of the pandemic (median days [IQR], 14 [8, 34] vs. 11 [5, 19]; *p* < 0.001) (Figure 3). Additionally, the duration of ICU length of stay among patients requiring intensive care treatment was shorter during the second wave of the pandemic (median days [IQR], 34 [19, 61] vs. 19 [11, 35], *p* = 0.001). A higher proportion of patients received antibiotic treatment during the first compared to the second wave (66% [*n* = 114] vs. 45% [*n* = 148]; *p* < 0.001). Hydroxychroloquin, lopinavir/ritonavir, tocilizumab [17], and the anti-adrenomedullin antibody adrecizumab [18] were only used during the first wave (Supplementary Materials Figure S2 and Table S2). Convalescent plasma was administered to six patients (3%) during the first wave and eight patients (2%) during the second wave. Remdesivir was primarily administered within clinical trials during the first observation period and, following licensure, increasingly used during the second observation period (6% [*n* = 10] vs. 15% [*n* = 49]). Dexamethasone was only administered to COVID-19 patients during the second wave of the pandemic. In total, 81 (16%) patients died during the entire study period. Of those, 27 deaths occurred during the first wave and 54 during the second wave, reflecting a case fatality rate of 16% for both phases of the pandemic (*p* = 0.82). Among all hospitalized patients, mortality was 19% for the entire cohort without significant differences between the first and the second wave (18% [*n* = 27] vs. 20% [*n* = 53]; *p* = 0.61). The overall mortality of patients treated at the intensive care unit was 39% (*n* = 66) for the entire period without significant differences during the first versus the second wave (32% [*n* = 24] vs. 44% [*n* = 42]; *p* = 0.15). Likewise, in-hospital mortality of patients treated on regular wards did not differ between the two periods (4% [*n* = 3] vs. 7% [*n* = 11]; *p* = 0.56). Only one elderly patient died as an outpatient in our emergency department during the second wave, who was already terminally ill at presentation. Deceased patients did not differ in age between the two waves (median age [IQR]; 72 years [61, 70] vs. 69 years [60, 80]; *p* = 0.38) (Figure 2c). Presentation during the first versus the second wave of the pandemic was not associated with increased in-hospital mortality in univariate (OR 1.1, 95% CI: 0.6–1.8; *p* = 0.82) and multivariate logistic regression analysis, which was adjusted for sex, age <60 or ≥60, and ACCI (OR: 1.2; 95% CI: 0.7–2.0; *p* = 0.56) (Supplementary Materials Table S3).

## 4. Discussion

We directly compared the main epidemiological and clinical characteristics of COVID-19 patients who presented at our tertiary care center in northern Germany during the first and the second wave of the pandemic. One of the main findings is that while we did not observe significant differences in median age and overall comorbidities, COVID-19 patients hospitalized during the second wave had a significantly shorter duration of hospitalization and a lower rate of patients required admission to the intensive care unit, mechanical ventilation, vasopressor treatment, or renal replacement therapy. These findings may be a direct result of increased knowledge about the clinical management of COVID-19 and improved treatment strategies that were developed during the year 2020. In the first weeks of the pandemic, lopinavir-ritonavir and hydroxychloroquine were frequently used due to some promising reports but later did not show clinical benefit compared to standard of care in randomized studies [19,20]. After the RECOVERY study demonstrated that low-dose dexamethasone reduced mortality in hospitalized COVID-19 patients who require respiratory support [21], German guidelines were adjusted accordingly [22], and dexamethasone was widely used at our hospital during the second phase of the pandemic. Studies on the efficacy of remdesivir in hospitalized COVID-19 patients have shown conflicting results. While the results of the ACTT-I study suggest a reduction in time to clinical improvement and, in patients requiring supplemental oxygen but not ventilation, a survival benefit in patients after 14 and 28 days [23], the SOLIDARITY trial showed no such mortality benefit after 28 days [24]. At our hospital, remdesivir was primarily administered within clinical trials during the first observation period and, following licensure, increasingly used during the second observation period. In line with other studies [25], utilization of NIV rapidly increased during the second wave compared to the first wave as reports on the potential of NIV in the treatment of respiratory failure in COVID-19 patients emerged. This may have contributed to a reduced rate of patients requiring mechanical ventilation in our study cohort. Furthermore, a better understanding of the management of patients with COVID-19 was associated with a significantly lower rate of patients who were treated with antibiotics during the second versus the first wave. Another important finding of our study is the decreased rate of hospitalization among COVID-19 patients presenting at our hospital throughout the pandemic, which is in line with national surveillance data showing that in Germany fewer diagnosed COVID-19 patients were admitted to hospitals [26]. Better knowledge about risk factors for a severe clinical course may have facilitated a better selection of patients requiring hospital admission [27]. Importantly, various infection control measures, including universal masking of healthcare workers and routine RT-PCR-screening of all patients upon admission, were established at our center [28], as the knowledge of the particular SARS-CoV-2 transmission patterns increased during the early months of 2020 [29,30]. Thus, the rate of nosocomial infections significantly decreased throughout the study period and vulnerable patient groups were more efficiently protected from COVID-19.

A striking finding of our study is that despite the abovementioned improvements in the clinical management of COVID-19 patients, in-hospital mortality was persistently high throughout the entire study period. In total, 16% of all COVID-19 patients presenting at our hospital in the year 2020 and 20% of those admitted and not treated as outpatients died. In our cohort, older age and a higher comorbidity index but not presentation during the first versus second wave of the pandemic were independently associated with higher in-hospital mortality. The overall in-hospital mortality was similar to other cohort studies from Germany that reported case fatality rates of 14% to 24% for hospitalized patients [31,32,33,34,35] and 35% to 53% for patients admitted to the intensive care unit [35,36,37]. However, markedly diverse inpatient mortality rates due to COVID-19 have been reported across different countries [38]. The extreme straining of many healthcare systems surely has contributed to high mortality rates in some countries especially during the early phases of the pandemic [16,39,40,41]. Ticinesi et al. reported that while COVID-19 patients treated with COVID-19 at an Italian hospital hub during the first weeks of the pandemic were younger and had fewer comorbidities than those admitted between late March and early June 2020, mortality decreased from 27% to 22% [42]. Likewise, a large retrospective study analyzing the hospital course of 20,736 COVID-19 patients in the US recently demonstrated that in-hospital mortality rates decreased from 19% in March and April 2020 to 11% in September through November 2020 and that this difference persisted after accounting for age, sex, and comorbidities [43]. Of note, our hospital was never overwhelmed and there were sufficient resources for treating every patient with all therapeutic options of modern intensive care medicine at any time of the pandemic without the need for triage. This is reflected by relatively low hospital mortality during the first wave of the pandemic compared to healthcare settings with higher patient burden. Importantly, the abovementioned novel treatment strategies for COVID-19 did not result in a decrease in mortality among patients treated at our center. These findings highlight that in a real-life setting, sufficient access to conventional therapy for all patients has a much higher impact on hospital mortality than the increased use of novel therapeutic agents.

While we did not observe differences in the median age of patients presenting at either of the two periods, relatively more patients 80 years and older were treated at our center during the second period. This is in line with surveillance data from Germany that show an increased SARS-CoV-2 incidence among age groups 80 years and older during the end of the second wave of the pandemic, partly driven by outbreaks in local nursing and long-term care facilities [44]. This is in contrast to previous studies from other European countries, which have shown that patients hospitalized during the second wave were relatively younger [45,46]. Despite those age differences among hospitalized patients at different phases of the pandemic, it has been shown that >80% of deaths occurred in patients aged 80 years and older in most European countries in both the first and the second wave of the pandemic [47], which is also in line with our single-center findings.

Our study has important limitations. First, the monocentric study character may limit generalizability to other hospitals and especially to other healthcare systems. Importantly, our hospital is a tertiary referral center that provides care for patients with critical disease requiring intensive care and ECMO therapy. Thus, disease severity of COVID-19 patients and in-hospital mortality may be higher compared to other hospitals. Yet, this study design enabled us to directly compare patients treated in the identical clinical setting during different periods of the pandemic. Second, due to a lack of systematic outpatient follow-up examinations, we were only able to determine in-hospital morbidity and mortality but not, while unlikely, any complications that might have occurred after discharge from the hospital or the emergency department. Third, at the beginning of the pandemic, patients were treated as inpatients not only because of the severity of the disease but also for isolation purposes and due to uncertainties about the disease course. Additionally, patients requiring admission to the ICU may have been transferred to our center earlier in the disease course in the first compared to the second wave, when there was little experience in the management of COVID-19 patients with ARDS especially at smaller hospitals. Unfortunately, we do not have information on the onset of symptoms for all patients so we cannot exclude that patients may have presented at different disease stages during the different phases of the pandemic. These factors may have had an important impact on the reported differences in the duration of hospitalization between the first and the second wave. Lastly, we only included patients who presented at our center until the end of the year 2020, when the second wave was still ongoing. However, the observed findings are likely to be attributable to the entire second wave of the pandemic. Importantly, the first infections with highly transmissible SARS-CoV-2 variants of concern were detected in Germany only during the last days of the year 2020 [48] and did thus not have an impact on our study results. However, it has been suggested that even during the first months of the pandemic genomic epidemiology of SARS-CoV-2 may have been associated with disease severity and may have had an impact on patient outcomes [49].

In the future, increased circulation of the variant of concern, increasing vaccination coverage, and novel treatment strategies, such as monoclonal antibodies, public health measures, and contact restrictions, will likely entail changes in the clinical characteristics of hospitalized COVID-19 patients. Future studies are needed to dynamically assess these changes throughout the evolving pandemic.

## 5. Conclusions

We demonstrate that even though no differences in age and comorbidities were observed between COVID-19 patients who presented during the first and second waves of the pandemic, the duration of hospitalization decreased, fewer nosocomial SARS-CoV-2 infections occurred, and a lower rate of patients was admitted to the intensive care unit during the second wave. Despite those improvements in the clinical management of COVID-19 patients, in-hospital mortality remained unaltered at a high level throughout the pandemic. These findings call for continuous efforts to prevent the transmission of SARS-CoV-2, to develop novel therapeutic options for patients with COVID-19, and to increase vaccination coverage.

## Figures and Tables

**Table 1:** Demographic information and comorbidities.

	All Patients	First Wave (27 February–28 July)	Second Wave (29 July–31 December)	*p*
Total	505	174	331	
Male, *n* (%)	292 (58)	117 (67)	175 (53)	0.002
Age, median (IQR)	58 (43, 72)	59 (46, 71)	58 (42, 73)	0.82
Age < 60, *n* (%)	268 (53)	88 (51)	180 (54)	0.03
Age 60–79, *n* (%)	176 (35)	72 (41)	104 (31)	
Age ≥ 80, *n* (%)	61 (12)	14 (8)	47 (14)	
ACCI, median (IQR)	2 (2, 4)	2 (1, 4)	2 (0, 4)	0.50

IQR, interquartile range; ACCI, age-adjusted Charlson Comorbidity Index.

**Table 2:** Course of disease.

	All Patients	First Wave (27 February–28 July)	Second Wave (29 July–31 December)	*p*
**Disease severity, *n* (%)**				
Mild	169 (33)	41 (24)	128 (39)	0.005
Moderate	76 (15)	27 (16)	49 (15)	
Severe	92 (18)	38 (22)	54 (16)	
Critical	168 (33)	68 (39)	100 (30)	
**Location of treatment, *n* (%)**				
Outpatient treatment	87 (17)	20 (11)	67 (20)	0.002
Regular ward	247 (49)	79 (45)	168 (51)	
ICU	171 (34)	75 (43)	96 (29)	
**Duration of hospitalization, median (IQR)**	11 (6–23)	14 (8–34)	11 (5–19)	<0.001
**Hospital course, *n* (%)**				
Nosocomial infection	28 (6)	24 (14)	4 (1)	<0.001
Transferred to our center	76 (15)	29 (17)	47 (14)	0.51
HFNC	65 (13)	28 (16)	37 (11)	0.13
NIV	47 (9)	8 (5)	39 (12)	0.009
Mechanical ventilation	123 (24)	56 (32)	67 (20)	0.004
Vasopressor treatment	136 (27)	60 (34)	76 (23)	0.006
Antibiotic treatment	262 (52)	114 (66)	148 (45)	<0.001
RRT	84 (17)	37 (21)	47 (14)	0.045
ECMO	50 (10)	13 (7)	37 (11)	0.21
Death	81 (16)	27 (16)	54 (16)	0.89

IQR, interquartile range; ICU, intensive care unit; HFNC, high-flow nasal cannula; NIV, non-invasive ventilation; RRT, renal replacement therapy; ECMO, extracorporeal membrane oxygenation.

## Data Availability

Data sharing does not apply to this article.

## Supplementary Materials

The following are available online at https://www.mdpi.com/article/10.3390/jcm10112274/s1, Figure S1: Comorbidities of COVID-19 patients, Figure S2: Timeline of usage of COVID-19 different therapeutics, Table S1: Immunosuppression of COVID-19 patients, Table S2: Specific treatment, Table S3: Multivariate logistic regression model.
