# Supplementary material for: Patient Characteristics and Clinical Course of COVID-19 Patients Treated at a German Tertiary Center during the First and Second Waves in the Year 2020
Source: J Clin Med. 2021 May 24;10(11):2274. doi: 10.3390/jcm10112274 (PMC8197386; doi:10.3390/jcm10112274)
Supplement: Supplementary file 1 [file jcm-10-02274-s001.zip › Table_S3.pdf]

Table S3. Multivariate logistic regression model

Variable	OR	95% CI	P
Presentation during first wave	1.2	0.7 – 2.0	0.56
Male sex	1.7	1.0 – 2.9	0.05
Age ≥ 60 years	2.5	1.3 - 5.0	0.009
ACCI	1.2	1.1 - 1.4	0.001

OR, odds ratio; CI, confidence interval; ACCI, age-adjusted Charlson Comorbidity Index
