# Supplementary material for: Patient Characteristics and Clinical Course of COVID-19 Patients Treated at a German Tertiary Center during the First and Second Waves in the Year 2020
Source: J Clin Med. 2021 May 24;10(11):2274. doi: 10.3390/jcm10112274 (PMC8197386; doi:10.3390/jcm10112274)
Supplement: Supplementary file 1 [file jcm-10-02274-s001.zip › Table_S1.pdf]

Table S1. Immunosuppression of COVID-19 patients

	**All patients**	**First wave** (Feb 27 - Jul 28)	**Second wave** (Jul 29 – Dec 31)
Total, n (%)	108 (21)	41 (24)	67 (20)
Steroids, n (%)	52 (10)	15 (9)	37 (11)
CNI/mTORI, n (%)	17 (3)	6 (3)	11 (3)
Anti-TNF-alpha, n (%)	6 (1)	0	6 (2)
MTX, n (%)	7 (1)	2 (1)	5 (2)
CD20 antibodies, n (%)	19 (4)	10 (6)	9 (3)
Chemotherapy, n (%)	40 (8)	20 (11)	20 (6)
Leukemia, n (%)	19 (4)	14 (8)	5 (2)
Lymphoma, n (%)	13 (3)	8 (5)	5 (2)
HIV, n (%)	2 (0)	0	2 (1)
Solid Organ Transplant, n (%)	26 (5)	7 (4)	19 (6)
Allogenic HSCT, n (%)	7 (1)	5 (3)	2 (1)

Immunosuppression of 505 patients with SARS-CoV-2 infections at our center during the first or second COVID-19 wave in the year 2020. CNI, calcineurin inhibitor; mTORI, mTOR-inhibitor ; MTX, metothrexate Anti-CD20; HIV, human immunodeficiency virus; HCT, hematopoietic stem-cell transplantation
