# Supplementary material for: Patient Characteristics and Clinical Course of COVID-19 Patients Treated at a German Tertiary Center during the First and Second Waves in the Year 2020
Source: J Clin Med. 2021 May 24;10(11):2274. doi: 10.3390/jcm10112274 (PMC8197386; doi:10.3390/jcm10112274)
Supplement: Supplementary file 1 [file jcm-10-02274-s001.zip › Table_S2.pdf]

Table S2. Specific treatment

	All patients	First wave (Feb 27 - Jul 28)	Second wave (Jul 29 – Dec 31)	P
Remdesivir, n (%)	59 (12)	10 (6)	49 (15)	0.002
Dexamethason, n (%)	95 (19)	0	95 (29)	<0.001
Convalescent plasma, n (%)	14 (3)	6 (3)	8 (2)	0.57
Hydroxychloroquin, n (%)	9 (2)	8 (5)	1 (1)	0.001
Tocilizumab, n (%)	3 (1)	3 (2)	0	0.04
Lopinavir/Ritonavir, n (%)	8 (2)	8 (5)	0	<0.001
Adrecizumab, n (%)	8 (2)	8 (5)	0	<0.001
