# Supplementary figures and images for: Patient Characteristics and Clinical Course of COVID-19 Patients Treated at a German Tertiary Center during the First and Second Waves in the Year 2020
Source: J Clin Med. 2021 May 24;10(11):2274. doi: 10.3390/jcm10112274 (PMC8197386; doi:10.3390/jcm10112274)

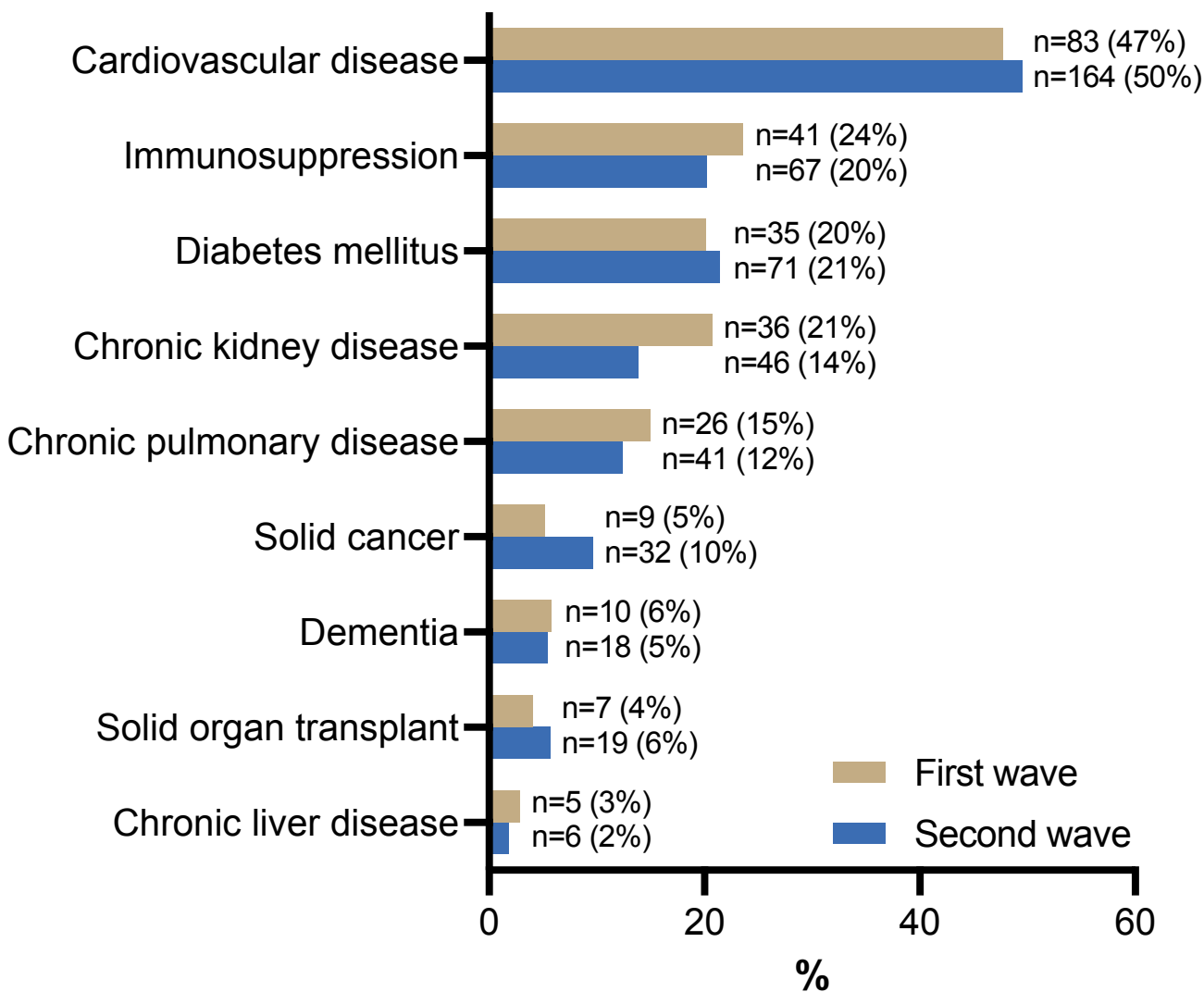

Supplement: Supplementary file 1 [file jcm-10-02274-s001.zip › Figure_S1.pdf]

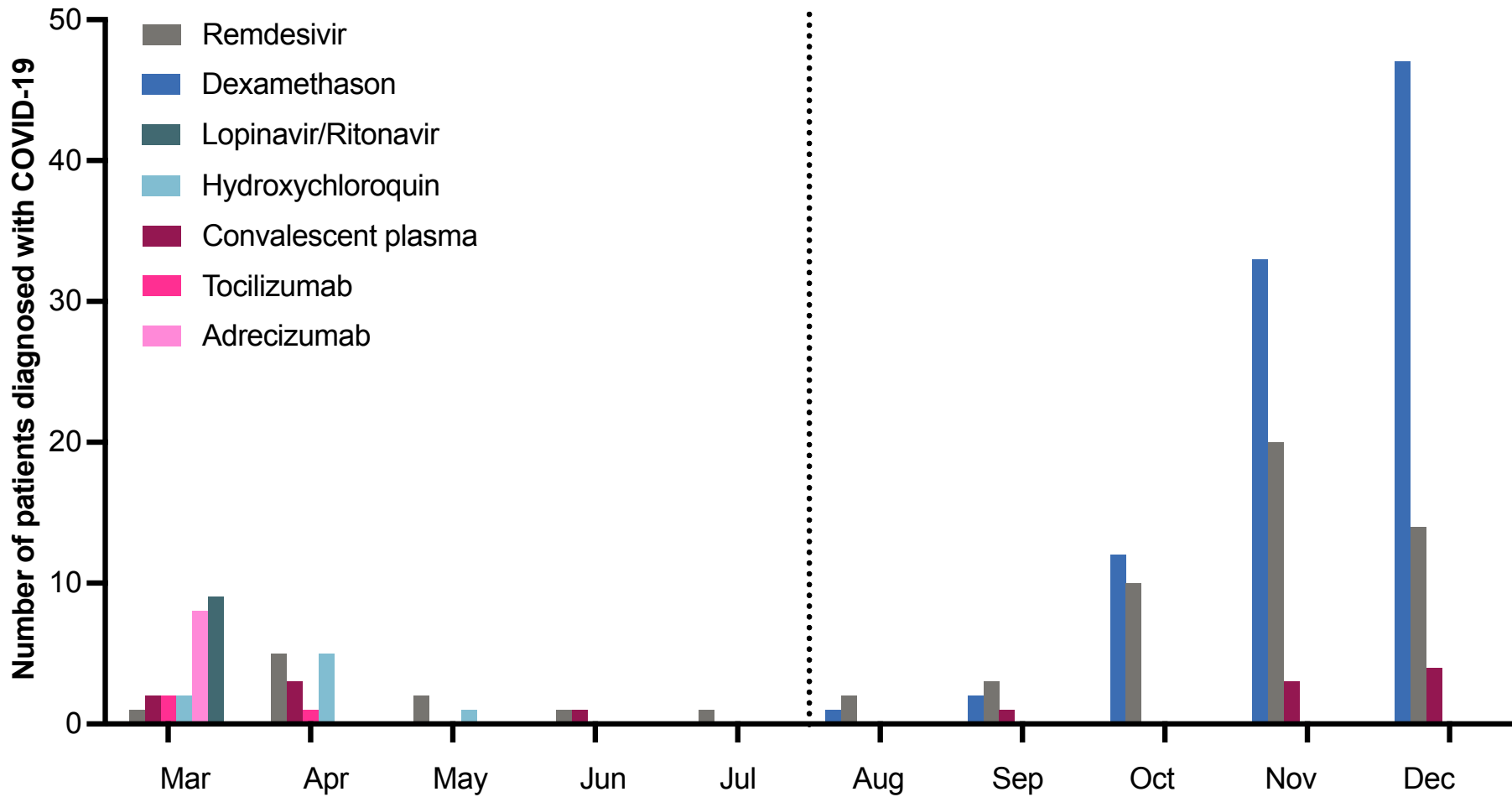

Supplement: Supplementary file 1 [file jcm-10-02274-s001.zip › Figure_S2.pdf]
